# Vitamin C alleviates LPS-induced myocardial injury by inhibiting pyroptosis via the ROS-AKT/mTOR signalling pathway

**DOI:** 10.1186/s12872-022-03014-9

**Published:** 2022-12-22

**Authors:** Pu Zhang, Meirong Zang, Zhenzhen Sang, Yunxia Wei, Yan Yan, Xiaohua Bian, Shimin Dong

**Affiliations:** 1 Department of Emergency, The Third Hospital of Hebei Medical University, Zi-Qiang Road No. 139, Shijiazhuang, 050051 Hebei China; 2 Department of Otolaryngology, The Third Hospital of Hebei Medical University, Shijiazhuang, Hebei China; 3 Department of Haematology, The Third Hospital of Hebei Medical University, Shijiazhuang, Hebei China

**Keywords:** Sepsis, Myocardial injury, Vitamin C, Pyroptosis, Reactive oxygen species (ROS)

## Abstract

**Background:**

The efficacy of vitamin C in sepsis remains controversial. Whether vitamin C can alleviate lipopolysaccharide (LPS)-induced myocardial injury by inhibiting pyroptosis has not been studied. This study aimed to evaluate the effects of vitamin C on LPS-induced myocardial injury in vitro.

**Methods:**

H9C2 cells were treated with indicated concentrations of LPS, and the cell viability was then assessed by CCK-8 assay. The levels of lactate dehydrogenase (LDH), CK-MB, IL-18 and IL-1β were examined by enzyme-linked immunosorbent assay (ELISA). The levels of intracellular reactive oxygen species (ROS) were measured using the fluorescent probe dichlorodihydrofluorescein diacetate (DCFH-DA). Western blot assays were conducted to determine the levels of the ROS-associated protein nicotinamide adenine dinucleotide phosphate oxidase 4 (Nox4) and pyroptosis-associated proteins, such as NOD-like receptor (NLR) family pyrin domain containing 3 (NLRP3), caspase-1 and gasdermin D (GSDMD). The AKT inhibitor MK-2206 was then applied to explore the signalling pathway. Finally, H9C2 cells were divided into the control group, LPS group, vitamin C + LPS group, and *N*-acetyl-l-cysteine (NAC) + LPS group. The intracellular ROS, levels of associated proteins, cell viability, and release of LDH, CK-MB, IL-18 and IL-1β were examined.

**Results:**

LPS decreased cell viability and induced ROS and pyroptosis in H9C2 cells in a dose-dependent manner. Moreover, LPS activated the AKT/mTOR pathway in H9C2 cells. The AKT inhibitor MK-2206 protected H9C2 cells from LPS-induced death by suppressing pyroptosis, without changing intracellular ROS level. Vitamin C significantly inhibited intracellular ROS and cell pyroptosis in LPS-treated H9C2 cells. Moreover, vitamin C suppressed the activation of the AKT/mTOR pathway.

**Conclusions:**

Our data suggest that vitamin C alleviates LPS-induced myocardial injury by inhibiting pyroptosis via the ROS-AKT/mTOR signalling pathway and thus provide novel insights into the prevention of sepsis-induced myocardial dysfunction.

**Supplementary Information:**

The online version contains supplementary material available at 10.1186/s12872-022-03014-9.

## Background

Sepsis is a clinical syndrome associated with fatal organ dysfunction caused by a systemic inflammatory response to infection and is an important cause of death in severe clinical patients [1, 2]. The heart, which is a vital organ in humans, is easily and rapidly affected during the development of sepsis [3]. Overall, myocardial injury occurs in more than 40–50% of cases of sepsis, and the mortality can exceed 50% [4]. Thus, a better pathophysiologic understanding of sepsis-induced myocardial injury and novel prevention strategies are urgently needed.

Multiple mechanisms have been proposed to be involved in the pathophysiologic processes of sepsis-induced myocardial injury, including persistent inflammatory responses [5, 6], energetic starvation [7], mitochondrial dysfunction [8] and oxidative stress [9]. However, recent evidence indicates that pyroptosis may be a main cause of heart failure and death [10]. Reactive oxygen species (ROS)-dependent pyroptosis has also been reported as an important pathway that mediates sepsis-induced myocardial injury [11]. Pathogen-associated molecular patterns (PAMPs), such as lipopolysaccharide (LPS), can induce ROS production, which can initiate NOD-like receptor (NLR) family pyrin domain containing 3 (NLRP3) inflammasome activation and form an inflammasome complex, leading to autocatalytic activation of caspase-1. Activated caspase-1 cleaves gasdermin D (GSDMD) to release its active N-terminal fragment, which forms pores on the plasma membrane, causing IL-1β and IL-18 release and pyroptosis [11, 12]. However, to the best of our knowledge, no studies have explored which signalling pathway participates in the process that ROS initiates NLRP3 and pyroptosis. AKT, known as protein kinase B (PKB), belongs to the serine/threonine kinase family and plays important roles in physiological and pathological processes. The AKT/mammalian target of rapamycin (mTOR) pathway is a critical cellular cascade in the cellular response to extracellular stimuli. It has been demonstrated that the AKT/mTOR signalling pathway is driven by ROS in LPS-induced myocardial injury [13]. However, whether the AKT/mTOR pathway participates in pyroptosis is unclear.

Vitamin C, also known as ascorbic acid, is a water-soluble antioxidant that can reduce the inflammatory response. The level of vitamin C in patients with sepsis is significantly decreased, and most patients exhibit vitamin C deficiency [14]. Shi-Jin et al. found that the intravenous administration of high-dose vitamin C at the early stage of sepsis could reduce the mortality of septic patients [15]. Another study found that the intravenous administration of vitamin C in combination with corticosteroids and thiamine is effective in preventing organ dysfunction and decreasing the mortality of patients with sepsis [16]. In vitro studies have demonstrated that vitamin C inhibits the expression of nicotinamide adenine dinucleotide phosphate oxidase 4 (Nox4) induced by inflammation, decreases the formation of ROS [17], and protects cells from oxidative damage [18]. It is believed that vitamin C exerts multiple effects in the treatment of patients with sepsis through its antioxidative and anti-inflammatory properties. However, whether vitamin C can alleviate LPS-induced myocardial injury by inhibiting pyroptosis has not been studied. Furthermore, several investigations have indicated that vitamin C administration does not exert a beneficial effect on patients with sepsis [19, 20]. Thus, the efficacy of vitamin C in sepsis remains controversial.

In this study, we hypothesize that vitamin C attenuates LPS-induced myocardial injury by inhibiting ROS-dependent NLRP3-mediated pyroptosis. We also explored whether the AKT/mTOR signalling pathway is involved in the process of ROS initiating pyroptosis. Our study aimed to obtain a new perspective on the pathophysiological process of sepsis-induced myocardial injury and to provide a novel prevention strategy.

## Materials and methods

### Cell culture

The rat cardiomyocyte cell line H9C2 was purchased from Procell Life Science & Technology, Wuhan, China. The cells were cultured in Dulbecco’s modified Eagle’s medium (DMEM) (Gibco, USA) supplemented with 10% foetal bovine serum (FBS) (Gibco, USA) and 1% penicillin‒streptomycin (Gibco, USA) in a humidified atmosphere containing 5% CO_2_ at 37 °C. Moreover, the culture media was replaced every 3 days. Cell passage was conducted once the cell density reached 80%. Trypsin–EDTA (0.25%) (GIBCO, USA) was used to digest the cells.

### Reagents

LPS (Sigma #L4391), adenosine 5'-triphosphate disodium salt hydrate (ATP) (Sigma #A6419), N-acetyl-L-cysteine (NAC) (Glpbio #GC11786) and vitamin C (Glpbio #GC12979) were dissolved in sterile deionized water. The AKT inhibitor MK-2206 (Glpbio #GC16304) was dissolved in dimethyl sulfoxide (DMSO). Anti-NLRP3 (#15101), anti-GSDMD (#93709), anti-phospho-AKT (#4060), anti-phospho-mTOR (#5536) and anti-β-actin (#3700) were obtained from Cell Signalling Technology. Anti-caspase-1 (#PA5-78915) was purchased from Invitrogen. Anti-Nox4 (ab133303) was obtained from Abcam.

### Treatment and grouping

To assess the cytotoxicity of LPS and to explore LPS-induced ROS and pyroptosis, H9C2 cells were divided into a control group and LPS treatment groups, which were exposed to different final LPS concentrations (0.1, 0.5 or 1.0 μg/mL) for 3 h. The cell culture media was then replaced, and 4 mM ATP was added for another 24 h. To confirm whether LPS plays a role through the AKT/mTOR pathway, H9C2 cells were divided into a control group, LPS group and MK-2206 + LPS group. The H9C2 cells in the MK-2206 + LPS group were pretreated with 0.5 μM MK-2206 for 1 h, whereas those in the control and LPS groups were pretreated with equal volumes of DMSO for 1 h. The H9C2 cells in the LPS and MK-2206 + LPS groups were then treated with 0.5 μg/ml LPS for 3 h. The cell culture media of all the groups was then replaced, and 4 mM ATP was added for another 24 h. To evaluate the protective role of vitamin C and NAC in LPS-induced myocardial injury, H9C2 cells were divided into a control group, LPS group, LPS + NAC group and LPS + vitamin C (LPS + Vc) group. The H9C2 cells in the LPS + NAC and LPS + Vc groups were pretreated with 2 μM NAC or 2 μM vitamin C for 1 h. All the groups except for the control group, cells were then treated with 0.5 μg/ml LPS for 3 h. The cell culture media of all the groups was subsequently replaced, and 4 mM ATP was added for another 24 h.

### Cell viability

H9C2 cells were seeded onto 96-well plates at a density of 5000 cells/well, cultured overnight at 37 °C and then subjected to the different treatments. Cell viability was assessed by a CCK-8 assay (HY-K0301, MCE, NJ, USA). Briefly, at the end of each treatment, 10 μL of CCK-8 solution was added to each well, and the plates were incubated at 37 °C in a humidified incubator with 5% CO_2_ for 4 h. The absorbance of each well was measured at 450 nm using a microplate reader (Flexstation® 3, Molecular Devices).

### Enzyme-linked immunosorbent assay (ELISA)

After the treatments, cell culture supernatants were cleared of cell debris by centrifugation at 1000 rpm for 20 min, and the concentrations of LDH (SEB864Ra, Cloud-Clone Corp, CCC, USA), CK-MB (SEA479Ra, Cloud-Clone Corp, CCC, USA), IL-1β (SEA563Ra, Cloud-Clone Corp, CCC, USA), and IL-18 (SEA064Ra, Cloud-Clone Corp, CCC, USA) were measured using ELISA kits according to the manufacturer’s instructions. The absorbance of each well at 450 nm was measured using a microplate reader.

### Measurement of ROS generation

The levels of intracellular ROS were measured with the fluorescent probe dichlorodihydrofluorescein diacetate (DCFH-DA, #S0033, Beyotime Biotechnology, China) according to the manufacturer’s instructions. Briefly, after the treatments, the cells were incubated with DCFH-DA (50 μM) at 37 °C for 20 min. The cells were then washed twice with phosphate-buffered saline (PBS), and DAPI (#C1002, Beyotime Biotechnology, China) was used to label the nuclei. Images were captured by fluorescence microscopy immediately after staining. The average fluorescence intensity was analysed using an image analysis system.

### Western blot analysis

Western blotting was performed with whole cell lysates extracted from cells after the treatments. Briefly, protein samples were separated using SDS‒PAGE and transferred to PVDF membranes. The membranes were blocked in 5% non-fat milk for 1 h at room temperature and then incubated overnight at 4 °C with primary antibodies (1:1000). The membranes were subsequently incubated with HRP-conjugated secondary antibodies (1:10,000, CST, USA) for 1 h at room temperature. The membranes were then washed, and protein signals were developed with enhanced chemiluminescence reagents (Thermo Scientific, MA, USA). The quantified protein expression level was normalized to β-actin. The Western blot analysis was repeated independently three times.

### Statistical analysis

The results are presented as the mean ± standard deviation (SD) values. Statistical analyses were performed using the Statistical Package for Social Sciences (SPSS) version 15.0 (IBM, Armonk, NY, USA). One-way ANOVA followed by Tukey’s test was performed to analyse the differences among experimental groups. *P* < 0.05 was considered to indicate statistical significance.

## Results

### LPS decreases viability and induces death in H9C2 cells

**Fig. 1:**
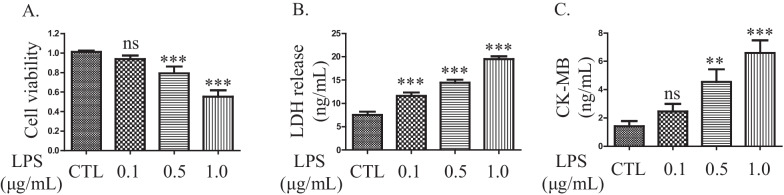
LPS decreases viability and induces death in H9C2 cells in a dose-dependent manner. **A** Cell viability assessment by CCK-8 assays. Data are presented as mean ± SD (n = 5). **B** LDH release assays. Data are presented as mean ± SD (n = 3). **C** CK-MB release assays. Data are presented as mean ± SD (n = 3). ***p < 0.001 compared with the control (CTL) group; **p < 0.01 compared with the CTL group; ns, *p* > 0.05 compared with the CTL group

To assess the cytotoxic effects of LPS, the rat cardiomyocyte cell line H9C2 was treated with serial concentrations of LPS for 3 h. Cell viability was measured by CCK-8 assays. A significant dose-dependent decrease in cell viability was observed in H9C2 cells: 1.01 ± 0.02 in the control group, 0.93 ± 0.04 in the 0.1 μg/mL LPS group, 0.79 ± 0.07 in the 0.5 μg/mL LPS group and 0.55 ± 0.07 in the 1.0 μg/mL LPS group (Fig. 1A). In addition, LDH release increased in a dose-dependent manner: 7.56 ± 0.66 ng/mL in the control group and 11.60 ± 0.75 ng/mL, 14.44 ± 0.93 ng/mL, and 19.51 ± 0.60 ng/mL in the groups exposed to increasing concentrations of LPS (Fig. 1B). Similar to the results for LDH release, CK-MB also exhibited a dose-dependent increase: 1.42 ± 0.37 ng/mL in the control group and 2.45 ± 0.54 ng/mL, 4.55 ± 0.89 ng/mL, and 6.59 ± 0.89 ng/mL in the groups exposed to increasing concentrations of LPS (Fig. 1C).

### LPS induces ROS production and pyroptosis in H9C2 cells

**Fig. 2:**
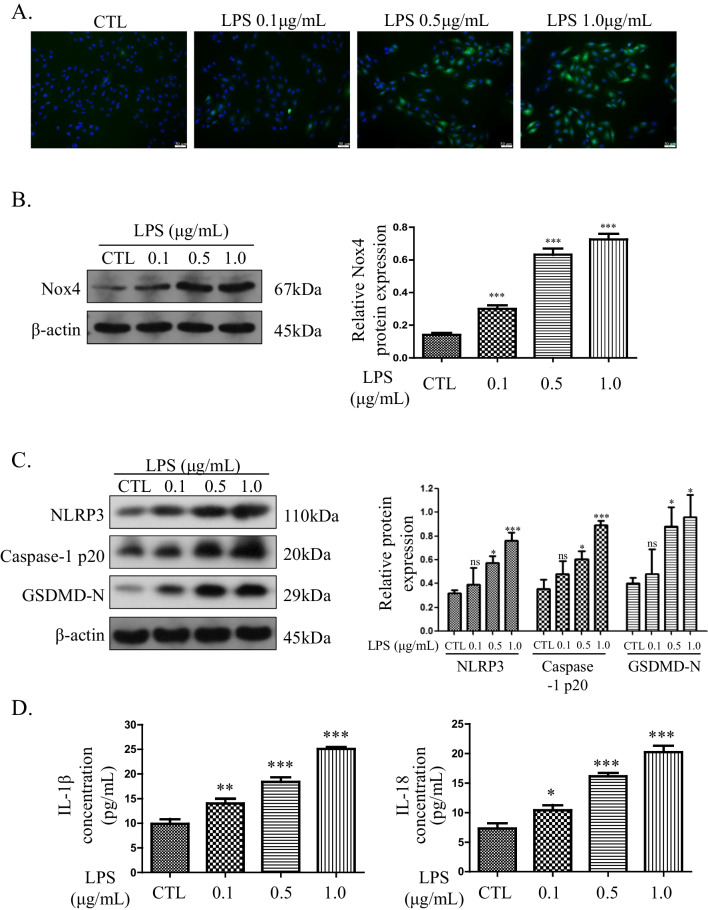
LPS induces ROS production and pyroptosis in H9C2 cells. **A** Intracellular ROS detected by DCFH-DA (magnification 200 ×). **B** ROS-associated protein Nox4 detected by western blotting. The cropped blots are shown on the left. The relative protein expression levels compared with the β-actin levels are shown on the right. Full-length blots are presented in Additional file 1: Figure S1A. **C** Pyroptosis-associated proteins detected by western blotting. The cropped blots are shown on the left. The relative protein expression levels compared with the β-actin levels are shown on the right. Full-length blots are presented in Additional file 1: Figure S1B. **D** Levels of IL-1β and IL-18 in the culture supernatants. Data are presented as mean ± SD (n = 3). *p < 0.05 compared with the control (CTL) group; **p < 0.01 compared with the CTL group; ***p < 0.001 compared with the CTL group; ns, *p* > 0.05 compared with the CTL group

To elucidate the reasons for the decreased cell viability and increased cell death caused by LPS, ROS and pyroptosis analyses were performed. As shown in Fig. 2A, the intracellular ROS level increased in a dose-dependent manner. Moreover, the expression of the ROS-associated protein Nox4 was upregulated in H9C2 cells by LPS, as determined by western blotting (Fig. 2B). The analysis of pyroptosis revealed that the expression of associated proteins was upregulated in H9C2 cells by LPS (Fig. 2C). Furthermore, the levels of the pyroptosis-associated inflammatory cytokines IL-1β (9.88 ± 0.96 pg/mL in the control group, 14.02 ± 1.00 pg/mL in the 0.1 μg/mL LPS group, 18.41 ± 0.94 pg/mL in the 0.5 μg/mL LPS group, and 25.09 ± 0.34 pg/mL in the 1.0 μg/mL LPS group) and IL-18 (7.31 ± 0.88 pg/mL, 10.42 ± 0.85 pg/mL, 16.15 ± 0.57 pg/mL, and 20.22 ± 1.09 pg/mL, respectively) were also positively correlated with the LPS concentration (Fig. 2D). In brief, LPS markedly induced ROS production and pyroptosis in a dose-dependent manner.

### LPS induces pyroptosis through the AKT/mTOR pathway in H9C2 cells

**Fig. 3:**
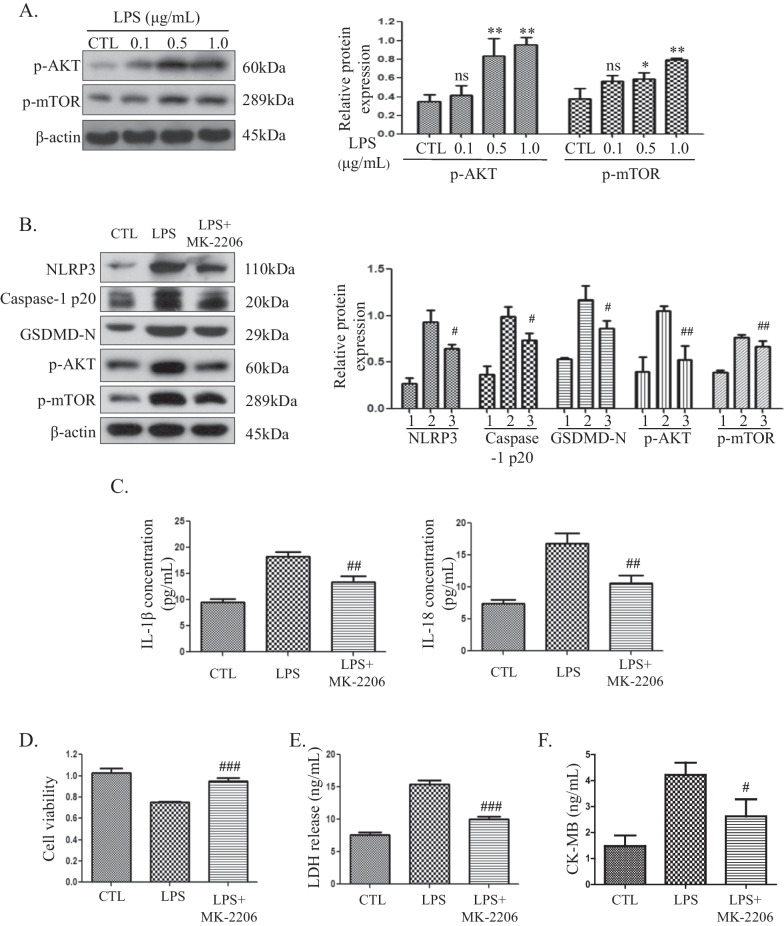
LPS induces pyroptosis through the AKT/mTOR pathway in H9C2 cells. **A** H9C2 cells were treated with the indicated concentrations of LPS, and the p-AKT and p-mTOR protein levels were detected by western blotting. The cropped blots are shown on the left. The relative protein expression levels compared with the β-actin levels are shown on the right. Full-length blots are presented in Additional file 1: Figure S2A. **B** Pyroptosis-associated proteins detected by western blotting. The cropped blots are shown on the left. The relative protein expression levels compared with the β-actin levels are shown on the right. 1: control (CTL) group, 2: LPS group, 3: LPS + MK-2206 group. Full-length blots are presented in Additional file 1: Figure S2B. **C** Levels of IL-1β and IL-18 in the culture supernatants. Data are presented as mean ± SD (n = 3). **D** Cell viability assessment by CCK-8 assays. Data are presented as mean ± SD (n = 5). **E** LDH release assays. Data are presented as mean ± SD (n = 3). **F** CK-MB release assays. Data are presented as mean ± SD (n = 3). *p < 0.05 compared with the control (CTL) group; **p < 0.01 compared with the CTL group; ns, *p* > 0.05 compared with the CTL group. #*p* < 0.05 compared with the LPS group; ##*p* < 0.01 compared with the LPS group; ###*p* < 0.001 compared with the LPS group

To gain further understanding of the mechanisms through which LPS mediates pyroptosis, we examined the effect of LPS on the AKT/mTOR pathway. H9C2 cells were exposed to serial concentrations of LPS for 3 h, and protein samples were subjected to western blot analysis of p-AKT and p-mTOR. An increase in p-AKT and p-mTOR expression was observed in LPS-treated H9C2 cells (Fig. 3A). LPS efficiently triggered the AKT/mTOR pathway in a dose-dependent manner. To explore whether the AKT/mTOR pathway participates in LPS-induced pyroptosis, the AKT inhibitor MK-2206 was applied. H9C2 cells were pretreated with 0.5 μM MK-2206 for 1 h and then treated with 0.5 μg/ml LPS for 3 h, and decreased expression of the pyroptosis-associated proteins NLRP3, caspase-1 and GSDMD was detected (Fig. 3B). Furthermore, the release of IL-1β and IL-18 was inhibited (Fig. 3C). The release of IL-1β in the LPS group (18.32 ± 0.78 pg/mL) was higher than that after MK-2206 pretreatment (13.37 ± 1.15 pg/mL). Moreover, the release of IL-18 also decreased from 16.85 ± 1.52 pg/mL to 10.62 ± 1.19 pg/mL after MK-2206 pretreatment. These results showed that the AKT/mTOR pathway plays roles in LPS-induced pyroptosis in H9C2 cells. To determine whether the MK-2206-mediated inhibition of pyroptosis was functional and protective in H9C2 cells, cell viability assays and LDH and CK-MB release analyses were performed. As shown in Fig. 3D, the cell viability of the LPS group was 0.75 ± 0.01, and MK-2206 pretreatment increased the viability to 0.95 ± 0.03. The release of LDH found for the LPS group (15.40 ± 0.65 ng/mL) was higher than that obtained after MK-2206 pretreatment (10.02 ± 0.41 ng/mL) (Fig. 3E). Moreover, the release of CK-MB also decreased from 4.21 ± 0.48 ng/mL to 2.62 ± 0.66 ng/mL after MK-2206 pretreatment (Fig. 3F). These findings suggest that the AKT inhibitor MK-2206 could prevent LPS-induced cell death via suppression of pyroptosis.

### Vitamin C protects H9C2 cells against LPS-induced cell death through the ROS-AKT/mTOR-pyroptosis pathway

**Fig. 4:**
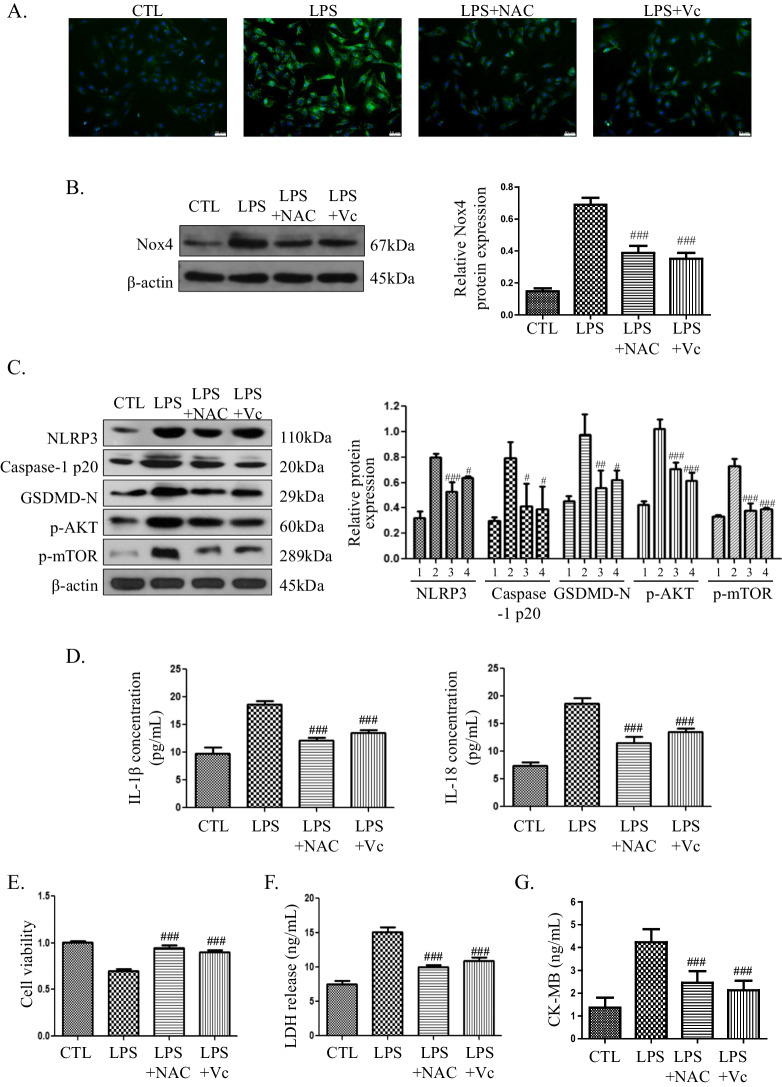
Vitamin C protects H9C2 cells against LPS-induced cell death through the ROS-AKT/mTOR-pyroptosis pathway. **A** Intracellular ROS level detected by DCFH-DA (magnification 200 ×). **B** Nox4 expression detected by western blotting. The cropped blots are shown on the left. The relative protein expression levels compared with the β-actin levels are shown on the right. Full-length blots are presented in Additional file 1: Figure S3A. **C** The expression of pyroptosis-associated proteins and p-AKT and p-mTOR was detected by western blotting. The cropped blots are shown on the left. The relative protein expression levels compared with the β-actin levels are shown on the right. 1: control (CTL) group, 2: LPS group, 3: LPS + NAC group, 4: LPS + Vc group. Full-length blots are presented in Additional file 1: Figure S3B. The full length membrane with edges visible of p-AKT was not provided because that the rest of the membrane belongs to a different experiment. **D** Levels of IL-1β and IL-18 in the culture supernatants. Data are presented as mean ± SD (n = 3). **E** Cell viability assessment by CCK-8 assay. Data are presented as mean ± SD (n = 5). **F** LDH release assays. Data are presented as mean ± SD (n = 3). **G** CK-MB release assays. Data are presented as mean ± SD (n = 3). #*p* < 0.05 compared with the LPS group; ##*p* < 0.01 compared with the LPS group; ###*p* < 0.001 compared with the LPS group

To evaluate whether vitamin C or NAC could protect H9C2 cells against LPS-induced cell death through the ROS-AKT/mTOR-pyroptosis pathway, H9C2 cells were pretreated with 2 μM vitamin C or 2 μM NAC for 1 h and then treated with 0.5 μg/ml LPS for 3 h. The LPS-induced ROS levels and Nox4 expression were decreased after pretreatment with NAC or vitamin C (Fig. 4A, B). Moreover, the AKT/mTOR pathway was inhibited, and as shown in Fig. 4C, the expression of p-AKT and p-mTOR was decreased. Pyroptosis was also inhibited, as shown by a decrease in pyroptosis-associated proteins (Fig. 4C) and reductions in IL-1β and IL-18 (Fig. 4D). The release of IL-1β in the LPS group (18.59 ± 0.74 pg/mL) decreased to 12.14 ± 0.49 pg/mL after NAC pretreatment and to 13.52 ± 0.47 pg/mL after vitamin C pretreatment. Moreover, compared with that in the LPS group (18.59 ± 1.08 pg/mL), the release of IL-18 was also reduced to 11.57 ± 1.10 pg/mL after NAC pretreatment and to 13.54 ± 0.58 pg/mL after vitamin C pretreatment. To evaluate the protective effect of NAC and vitamin C, a cell viability assay was performed. As shown in Fig. 4E, the cell viability of the LPS group was 0.70 ± 0.02, and higher viabilities of 0.95 ± 0.03 and 0.90 ± 0.02 were found in the LPS + NAC and LPS + Vc groups, respectively. Compared with that in the LPS group (15.12 ± 0.70 ng/mL), the release of LDH decreased to 10.01 ± 0.30 ng/mL after NAC pretreatment and to 10.93 ± 0.45 ng/mL after vitamin C pretreatment (Fig. 4F). Moreover, compared with that in the LPS group (4.23 ± 0.57 pg/mL), the release of CK-MB was also reduced to 2.46 ± 0.51 ng/mL in the LPS + NAC group and to 2.12 ± 0.41 pg/mL in the LPS + Vc group (Fig. 4G). The results indicated that vitamin C could protect H9C2 cells from LPS-induced death by inhibiting the ROS-AKT/mTOR-pyroptosis pathway.

## Discussion

Pyroptosis is a programmed cell death distinct from apoptosis, which is characterized by the formation of pores on the plasma membrane, cell swelling and osmotic lysis identified by observations of the cell morphology. The biochemical characteristics of the classical pyroptosis pathway are mainly marked by activation of caspase-1 and GSDMD and the release of the proinflammatory factors IL-1β and IL-18. Pyroptosis plays a crucial role in the dysregulation of inflammatory/immune responses in sepsis [21, 22]. In the present study, we found that LPS induced myocardial injury and activated ROS and pyroptosis in a dose-dependent manner in vitro (Figs. 1, 2). Our results further demonstrated that ROS and pyroptosis participated in LPS-induced myocardial injury.

ROS activate the AKT/mTOR signalling pathway in diabetic nephropathy [23]. During the process of hyperglycaemia-induced glomerular mesangial cell proliferation, excessive ROS could increase the expression of p-AKT [24]. Therefore, the AKT/mTOR signalling pathway can be upregulated by ROS in some pathological processes. However, a previous study showed that docosahexaenoic acid (DHA) increases the expression of p-AKT by reducing the production of ROS and alleviated pyroptosis-related hepatic ischaemia reperfusion injury [25]. Another study showed that in duck renal tubular epithelial cells, cadmium and molybdenum increases the ROS levels, downregulates p-AKT expression, and upregulates pyroptosis-related factors [26]. The different roles of ROS and the AKT pathway may be cell specific and related to different physiological conditions [25]. To our knowledge, no studies have explored whether the AKT/mTOR signalling pathway participates in the process of LPS-induced myocardial pyroptosis. In the present study, LPS significantly increased the expression of p-AKT and p-mTOR, which are the activated forms of AKT and mTOR, respectively. In addition, pretreatment with the AKT selective inhibitor MK-2206 decreased the expression of p-AKT and p-mTOR and protected H9C2 cells from LPS-induced pyroptosis without changing the expression of Nox4 and ROS production (Additional file 1: Fig. S4). These data suggest that LPS induces myocardial pyroptosis through the AKT/mTOR pathway, whereas ROS may be upstream of the AKT/mTOR pathway.

Vitamin C, as an antioxidant and an anti-inflammatory compound, is a safe, effective, and economical therapeutic approach for many diseases, such as cancer [27], ischaemia‒reperfusion injury and severe infection [28]. However, the efficacy of vitamin C in sepsis treatment remains controversial [19, 20]. It has been reported that vitamin C exerts an advantageous effect on the heart in septic patients [29], but whether vitamin C can alleviate LPS-induced myocardial injury by inhibiting pyroptosis has not been studied. In our present study, pretreatment with vitamin C and NAC (as a positive control) significantly reduced the expression of Nox4 and the production of ROS in H9C2 cells with LPS-induced myocardial injury. Our results also showed that vitamin C and NAC could inhibit the activation of the AKT/mTOR pathway and pyroptosis. Therefore, our study confirms the protective effect of vitamin C in LPS-induced myocardial injury in vitro. More importantly, these data suggest that the AKT/mTOR pathway is activated by ROS during LPS-induced pyroptosis. Thus, vitamin C could prevent myocardial cells from LPS-induced death through the ROS-AKT/mTOR-pyroptosis pathway in sepsis.

Although our findings provide a new possible pathological mechanism of sepsis-induced myocardial injury and data that support the preventive effect of vitamin C, this study has several limitations. (1) We only used pyroptosis-related proteins (NLRP3, caspase-1, GSDMD, IL-1β and IL-18) to identify the participation of pyroptosis but did not confirm this finding through a morphological analysis. Therefore, we will perform HE staining and electron microscopy to confirm pyroptosis-associated morphological changes, such as cell swelling and pore formation. (2) Only an in vitro model was used in our study, and the protective effect and mechanisms of vitamin C in LPS-induced myocardial injury should be further elucidated in a septic animal model. (3) The relationship of ROS and AKT/mTOR signalling pathway in LPS-induced pyroptosis was only studied using vitamin C and the AKT inhibitor MK-2206, which is not sufficient. Using an mTOR inhibitor and inhibiting the expression of key genes (such as Nox4, AKT, and GSDMD) by shRNA should also be used to confirm the upstream and downstream relationships.

## Conclusions

Our results indicate that LPS could increase the levels of Nox4 and ROS. The AKT/mTOR signalling pathway could be upregulated by ROS, and the ROS-AKT/mTOR signalling pathway participates in LPS-induced pyroptosis in H9C2 cells. Pretreatment with vitamin C alleviates LPS-induced myocardial injury by inhibiting pyroptosis via the ROS-AKT/mTOR signalling pathway. The present study provides new insight into the pathophysiology of sepsis-induced myocardial injury as well as a potential prevention strategy.

## Supplementary Information

**Additional file 1.** Supplementary Figures.

## Data Availability

The datasets used and analysed during the current study are available from the corresponding author upon reasonable request.

## Abbreviations

LPS: Lipopolysaccharide
LDH: Lactate dehydrogenase
ELISA: Enzyme-linked immunosorbent assay
ROS: Reactive oxygen species
DCFH-DA: Dichlorodihydrofluorescein diacetate
NAC: *N*-acetyl-l-cysteine
PAMPs: Pathogen-associated molecular patterns
NLR: NOD-like receptor
NLRP3: NOD-like receptor (NLR) family pyrin domain containing 3
GSDMD: Gasdermin D
DMEM: Dulbecco’s modified Eagle’s medium
FBS: Foetal bovine serum
ATP: Adenosine 5′-triphosphate disodium salt hydrate
PBS: Phosphate-buffered saline
SD: Standard deviation
SPSS: Statistical package for social sciences
DAMPs: Damage-associated molecular patterns
PKB: Protein kinase B
DHA: Docosahexaenoic acid
Nox4: Nicotinamide adenine dinucleotide phosphate oxidase 4
