# Supplementary material for: Vitamin C alleviates LPS-induced myocardial injury by inhibiting pyroptosis via the ROS-AKT/mTOR signalling pathway
Source: BMC Cardiovasc Disord. 2022 Dec 22;22:561. doi: 10.1186/s12872-022-03014-9 (PMC9783737; doi:10.1186/s12872-022-03014-9)
Supplement: Supplementary file 1 — Additional file 1. Supplementary Figures. [file 12872_2022_3014_MOESM1_ESM.doc]

**Supplementary Figure 1**


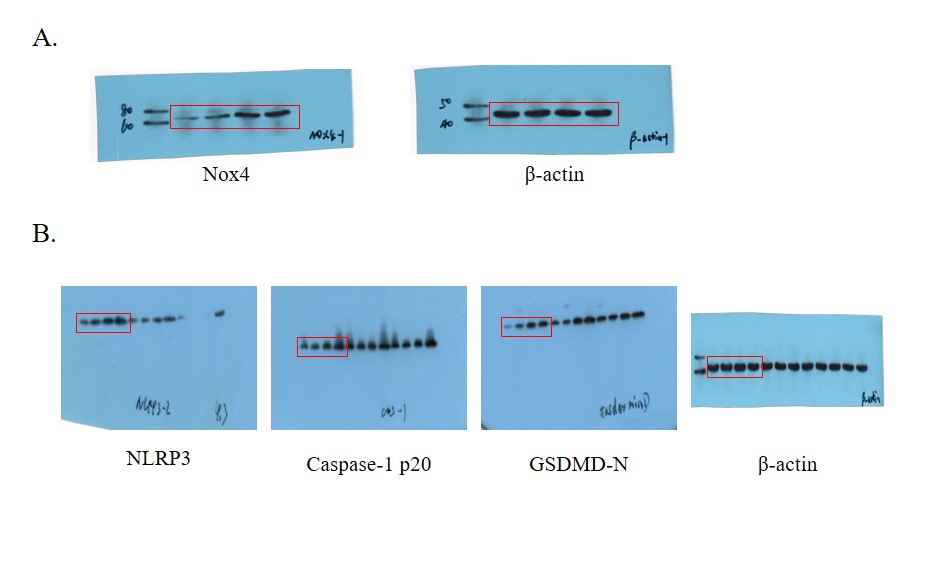


**Supplementary Figure 1.** Original western blot images of target proteins. (A) The original bands of western blot in Figure 2B. (B) The original bands of western blot in Figure 2C.

**Supplementary Figure 2**


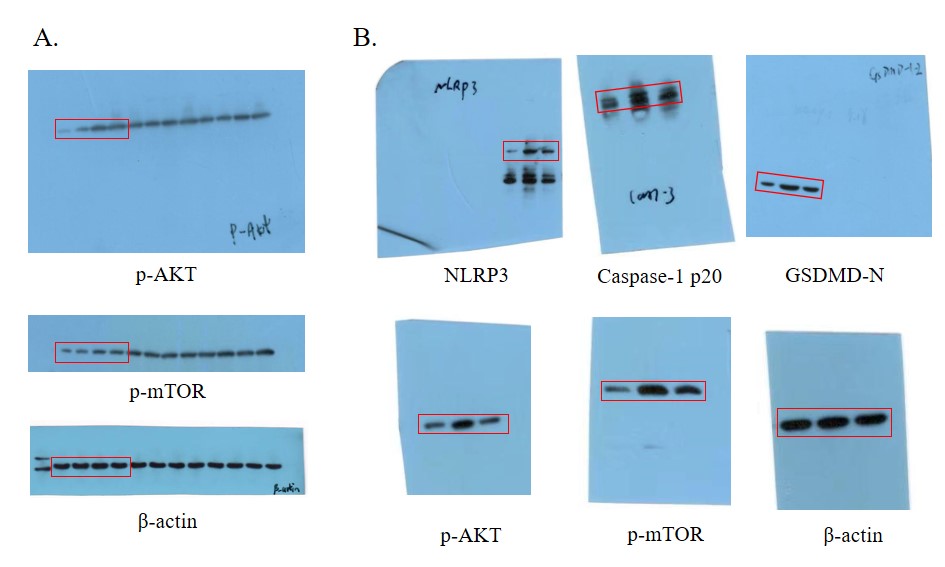


**Supplementary Figure 2.** Original western blot images of target proteins. (A) The original bands of western blot in Figure 3A. The original bands of β-actin in Figure 3A and Figure 2C are the same. (B) The original bands of western blot in Figure 3B.

**Supplementary Figure 3**


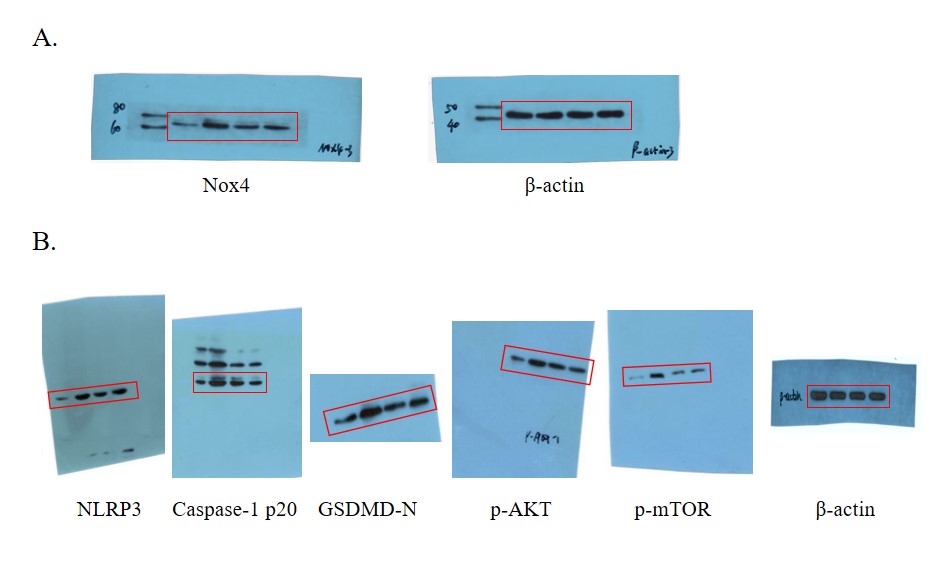


**Supplementary Figure 3.** Original western blot images of target proteins. (A) The original bands of western blot in Figure 4B. (B) The original bands of western blot in Figure 4C. The full length membrane with edges visible of p-AKT was not provided because that the rest of the membrane belongs to a different experiment.

**Supplementary Figure 4**

**
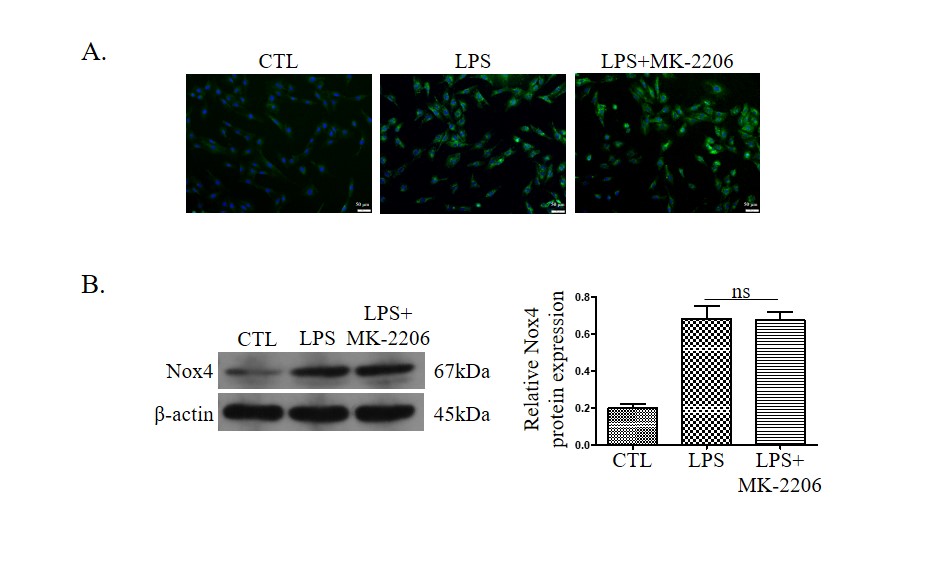
**

**Supplementary Figure 4.** (A) The intracellular ROS detected by DCFH-DA (magnification 200x). (B) ROS-associated protein Nox4 detected by western blot. The cropped blots are shown in the left. The relative protein expression levels compared to β-actin are shown in the right. Full-length blots are presented in Supplementary Figure 5.

**Supplementary Figure 5**

**
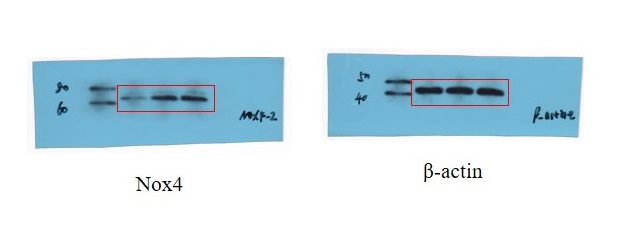
**

**Supplementary Figure 5.** The original bands of western blot in Supplementary Figure 4B.
